# Misinformation interventions and online sharing behaviour: lessons learned from two pre-registered field studies

**DOI:** 10.1098/rsos.251377

**Published:** 2025-11-26

**Authors:** Jon Roozenbeek, Jana Lasser, Malia Marks, Tianzhu Qin, David Garcia, Beth Goldberg, Ramit Debnath, Sander van der Linden, Stephan Lewandowsky

**Affiliations:** ^1^ Department of Psychology, University of Cambridge, Cambridge, UK; ^2^ Institute for Interactive Systems and Data Science, Graz, Austria; ^3^ Centre for Human-Inspired AI, University of Cambridge, Cambridge, UK; ^4^ University of Konstanz, Konstanz, Germany; ^5^ Google LLC, New York, NY, USA; ^6^ School of Psychological Science, University of Bristol, Bristol, UK

**Keywords:** misinformation, inoculation theory, intervention, field study, null results

## Abstract

The spread of misinformation on social media continues to pose challenges. While prior research has shown some success in reducing susceptibility to misinformation at scale, how individual-level interventions impact the quality of content shared on social networks remains understudied. Across two pre-registered longitudinal studies, we ran two Twitter/X ad campaigns, targeting a total of 967 640 Twitter/X users with either a previously validated ‘inoculation’ video about emotional manipulation or a control video. We hypothesized that Twitter/X users who saw the inoculation video would engage less with negative-emotional content and share less content from unreliable sources. We do not find evidence for our hypotheses, observing no meaningful changes in posting or retweeting post-intervention. Our findings are most likely compromised by Twitter/X’s ‘fuzzy matching’ policy, which introduced substantial noise in our data (approx. 7.5% of targeted individuals were actually exposed to the intervention). Our findings are thus probably the result of treatment non-compliance rather than ‘true’ null effects. Importantly, we also demonstrate that different statistical analyses and time windows (looking at the intervention’s effects over 1 h versus 6 h or 24 h, etc.) can yield different and even opposite significant effects, highlighting the risk of interpreting noise from field studies as signal.

## 1. Introduction

The spread of online misinformation has continued to pose a challenge for governments, tech companies, educators and researchers [1–3]. Interventions to counter information can occur at the system level (e.g. changing recommender algorithms) or at the individual level [4]. Within the latter realm, *refutational* approaches to tackling misinformation, such as debunking and fact-checking, are generally effective at reducing misbeliefs [5], but they also suffer from several limitations [6]. For example, fact checks on social media often do not reach the people who are most vulnerable to misinformation [7], and even if successful, misinformation can continue to reside in memory post-correction, a phenomenon known as the ‘continued influence effect’ [8].

In contrast, researchers have therefore explored ways to reduce the probability that people are persuaded by misinformation in the first place, an approach known as *prebunking* [9,10]. The most prominent method used to prebunk misinformation is rooted in inoculation theory [10–12]. Inoculation theory posits that it is possible to build psychological resilience against future unwanted persuasion attempts by pre-emptively exposing people to a ‘weakened dose’ of misinformation consisting of a forewarning of an impending attack on one’s belief as well as a pre-emptive refutation of the impending misinformation [13]. In recent years, researchers have especially focused on inoculating people against the manipulation techniques and tropes that underlie misinformation, such as logical fallacies, emotional manipulation and conspiratorial reasoning. The advantage of this approach is that people are inoculated against a broad range of content. Interventions that inoculate people against manipulation techniques commonly used in misinformation include games [14–17], videos [18–20] and text-based infographics [21,22].

A pressing open question in inoculation research is how inoculation interventions affect people’s behaviour on social media [9,23]. This is important because, ideally, misinformation interventions do not only target competences (such as the ability to correctly identify misleading content) but also behaviour (such as the sharing of misleading content with other people). In a field study on YouTube, Roozenbeek *et al*. [20] found that watching an inoculation video as a YouTube ad significantly boosted YouTube users’ ability to correctly identify manipulation techniques in news headlines. However, they were unable to look at behaviour, in the sense that they could not test if the inoculation ads reduced the probability of someone watching videos that contain false or misleading content, because social media companies are often unwilling to share such behavioural data [24]. More broadly, with some exceptions (see [25–29]), field studies that look at how misinformation interventions affect people’s behaviour online remain relatively scarce [4].

## 2. The present research

**Figure 1.**
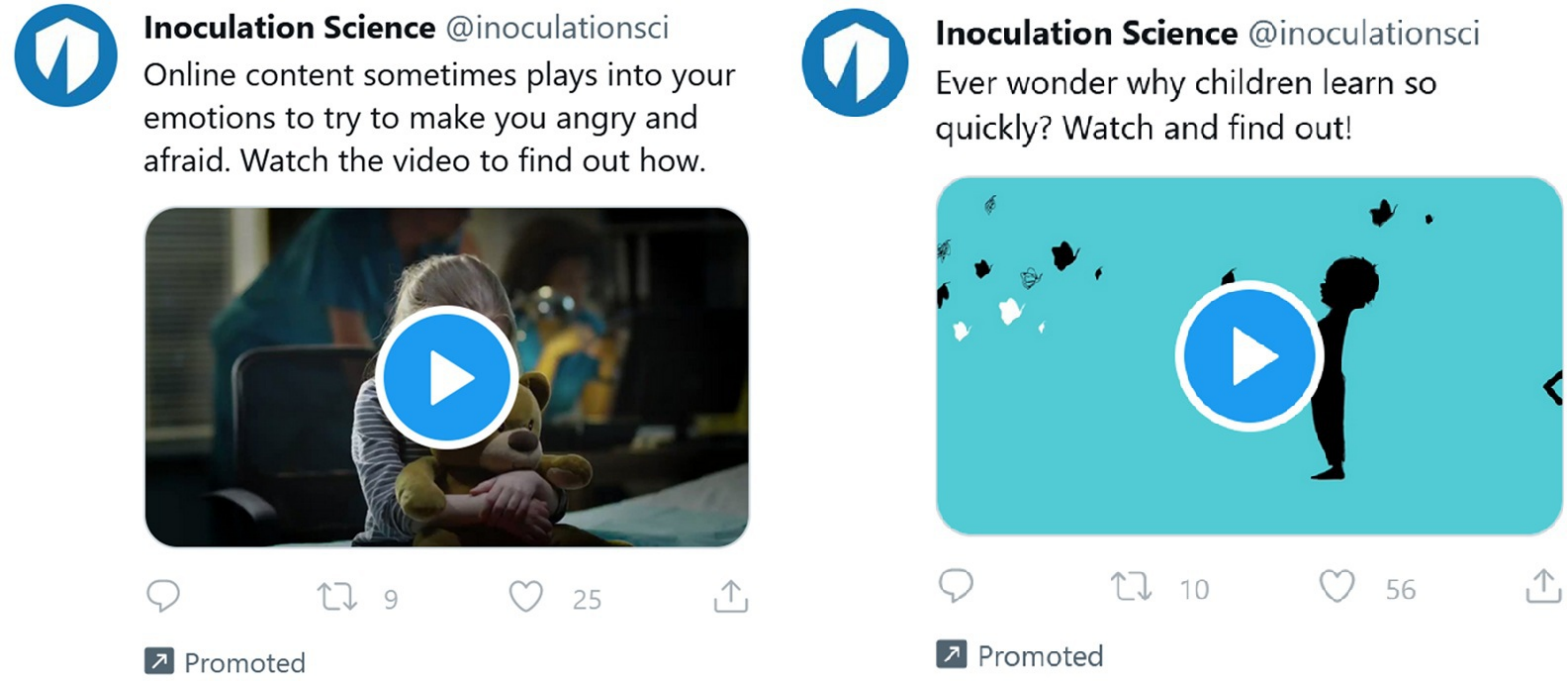
Examples of a Twitter/X ad with the inoculation intervention (left) and the control video (right).

We conducted two ecologically valid field studies, testing how inoculation interventions influence what people post and share on social media. We ran two separate ad campaigns on Twitter/X using a previously validated video-based inoculation intervention about emotional manipulation [20]. We chose this intervention because of its robustness in laboratory studies: the effect of this specific inoculation video on improving people’s ability to correctly evaluate emotionality in social media content has been extensively and successfully replicated [16,30,31]. Specifically, we showed a 30 s inoculation video as an advertisement to US Twitter/X users who had previously shared misinformation. Another group of Twitter/X users (matched to the treatment group in terms of demographics and past sharing behaviour) was shown a control video of about the same length about children’s learning abilities. We created a Twitter/X account, @inoculationsci, specifically for the purpose of running these ad campaigns. See figure 1 for an overview of the ads as they were shown on Twitter/X. Our data, analysis scripts, item sets and further electronic supplementary material can be found on our OSF page (https://osf.io/3whuy/).

Because the inoculation video shows viewers how to recognize emotionally manipulative content (and makes them better able to do so, see [20]), we expected that Twitter/X users who saw the video (i.e. the treatment group) would engage less with negative-emotional content and content from low-quality sources, both in terms of what they post themselves and what they retweet or share with others, compared with a control group of Twitter/X users who did not see the video. Specifically, we pre-registered the following hypotheses:

**H1**: Participants who watched the ‘emotional language’ video on Twitter/X post fewer tweets containing text with negative-emotional text (fear, anger) in their tweets after seeing the video, compared with a control group.
**H2**: Participants who watched the ‘emotional language’ video on Twitter/X retweet significantly fewer negative emotions after seeing the video, compared with a control group.
**H3**: Participants who watched the ‘emotional language’ video on Twitter/X post fewer tweets of sources containing low-quality news content, compared with a control group.
**H4**: Participants who watched the ‘emotional language’ video on Twitter/X retweet fewer sources containing low-quality news content, compared with a control group.

For study 1 only, we also conducted a follow-up campaign, in which individuals who were previously presented with either an inoculation video or a control video were asked to click a link to a Qualtrics survey. This survey contained an item-rating task where people were asked to evaluate a series of manipulative and neutral (non-manipulative) social media posts. This task provides a measure of ‘veracity discernment’, or how good people are at distinguishing between manipulative and neutral social media content. This item-rating task is the same as the one from studies 1 and 6 in Roozenbeek *et al*. [20]. We hypothesized that Twitter/X users who had been targeted with an inoculation video would perform significantly better at this item-rating task (i.e. have significantly higher veracity discernment) than users who had seen a control video. This follow-up survey had a very low response rate and yielded a total of 68 valid responses (table 1 and electronic supplementary material, table S1).

**Table 1.** Overview of samples and numbers of targeted Twitter/X users in each study (studies 1 and 2). Note: ‘unknown’ means that the number of targeted Twitter/X users is not available due to the user list being out of date as of the time of writing.

dataset	max. sample	targeted sample	impressions	video views	responses
**birdwatch**					
seed sample	4963	3829			
**ad campaigns**					
total	967 640		245 863	140 892	
study 1 (treatment)	100 000	unknown	47 569	29 456	
study 1 (control)	100 000	unknown	55 269	33 087	
study 2 (treatment)	323 993	155 300	70 700	39 067	
study 2 (control)	323 993	155 800	72 325	39 282	
**survey study (study 1 only)**					
treatment	100 000		132 770		41/118
control	100 000		187 575		29/94

## 3. Data and methods

### 3.1. Sample and procedure

To seed our user sample, we recruited Twitter/X users based on (what at the time was called) the Twitter Birdwatch dataset (https://web.archive.org/web/20220117040009/https://twitter.github.io/birdwatch/contributing/download-data/). We included users in the seed group who had at least one of their tweets classified as misinformation. To achieve this, we first obtained all tweets that were classified as misinformation on Birdwatch at least once and removed all non-English-language tweets. This resulted in 4963 unique users. We composed our final user group from followers of the users of the initial seed list by scraping the profiles of the 1000 most recent followers of these 4963 seed users. We successfully scraped the followers of 3829 out of 4963 seed users. After removing all user accounts with 100 or fewer followers, users with a bot score of greater than 0.8 and Twitter/X accounts flagged as ‘protected’, we ended up with a total of 967 640 user accounts. Out of these user accounts, 200 000 randomly selected accounts were used for study 1 (i.e. 100 000 per experimental condition).

We used the remaining user accounts for study 2, splitting the sample evenly into a treatment and control groups. Users in the treatment and control groups were matched based on their number of followers at the time when we scraped their profiles (September 2022). In total, study 2 included 323 993 users in each group. We uploaded these lists of usernames to the Twitter/X Adspace, resulting in a final sample that was available for ad targeting of approximately 155 300 users in the treatment group and 155 800 users in the control group. At the time, Twitter/X employed a so-called ‘fuzzy matching’ algorithm for privacy reasons, which means that a random 30% of uploaded users were actually targeted with the ads. Furthermore, table 1 shows that about 25% of users were shown a video (245 863 impressions on a sample of 967 640). This means that about 7.5% of our sample could have potentially benefited from the intervention (i.e. approx. 25% of approx. 30%). We therefore do not know exactly which users were exposed to the intervention and are working with extremely noisy data. The pilot study was launched at 07.00 ECT (East Coast time, USA) on 15 September 2021, and the full study at 07.00 ECT on 29 September 2022.^1^ Both campaigns cost a total of US$15 562.72 in ad expenditures. Table 1 shows an overview of the number of targeted Twitter/X users and the number of impressions, video views and survey responses per study.

### 3.2. Measures and analyses

We use two main outcome measures in this study. First, we look at emotional language use, specifically the probability of a tweet conveying a certain distinct emotion (*list*: anger, fear, sadness, disgust, pessimism; anticipation, joy, surprise, optimism, love, trust), as determined by a RoBERTa model trained on the SemEval 18 dataset [32]. Second, we look at news sharing quality, i.e. the average trustworthiness score of links to news sites shared, as determined by the NewsGuard database as of 13 September 2022 [33]. In line with our pre-registration, we explore both participants’ own posting behaviour and what they share with others (i.e. through retweets and exploratory analyses for replies and quote tweets).

In our pre-registration, we hypothesized that the difference between groups in negative emotion use is different from 0. For the full study, we use bootstrapping to generate confidence intervals to test this hypothesis and conduct a permutation test to see if between-group differences are significant at alpha = 0.01. Furthermore, we use bootstrapping to compare the trustworthiness scores of links with news sources shared pre- and post-intervention start time between groups. We also pre-registered a series of exploratory analyses to see whether the effects are moderated by gender and age, tweet engagement (likes, retweets, replies), the number of followers, bot score and organization score. Finally, we also look at the ‘sadness’ and toxicity of content as exploratory outcome variables. For study 1, we do not conduct statistical tests but instead rely on descriptive observations, as this was a pilot study.

### 3.3. Non-pre-registered analyses

To supplement the analyses above, we used a Difference-in-Difference (DiD) and a causal Bayesian inference approach to validate the findings from our online experiments. These analyses were not pre-registered. For the DiD analysis, we used various time windows around the intervention start (e.g. comparing the 24 h before the intervention with the 24 h after, 12 h before versus 12 h after, and so on). We do this because previous research has used various and sometimes non-pre-registered time windows to look at intervention effects [27,29]. This approach risks reporting significant findings for arbitrary time windows: without good theoretical predictions for how long intervention effects should last, selectively reporting results for specific time windows may not be best practice. The DiD framework is a quasi-experimental method that isolates the causal impact of an intervention by leveraging a natural counterfactual. By comparing changes in sentiment over time between treated and control groups, DiD eliminates time-invariant confounders and accounts for unobserved heterogeneity that could bias simpler pre–post comparisons. We specified our model as follows:


$$sentiment=\beta_{0}+\beta_{1}\cdot time+\beta_{2}\cdot treated+\beta_{3}\cdot \left(time\times treated\right)+\;\varepsilon ,$$

where sentiment represents the specific emotional response of interest, time is a binary indicator for post-intervention observations, treated is a binary variable denoting exposure to the intervention, (time × treated) captures the interaction effect measuring the differential change in sentiment due to the intervention and $\beta_{0}$, $\beta_{1}$, $\beta_{2}$, $\beta_{3}$ and ε are the intercept term, coefficients for each variable and error term, respectively. Specifically, $\beta_{0}$ represents the baseline sentiment in the control group before the intervention; $\beta_{1}$ captures the change in sentiment for the control group after the intervention; $\beta_{2}$ captures the pre-intervention difference in sentiment between the treated and control groups; and $\beta_{3}$, the coefficient of interest, estimates the DiD effect. This term isolates the treatment effect by subtracting the pre–post difference in sentiment for the control group from the pre–post difference in sentiment for the treated group. The calculation of $\beta_{3}$ is followed by the mathematical formulae below:


$$\begin{array}{rl} \beta_{3} & =\left({sentiment}_{time=1,\;treated=1}-{sentiment}_{time=0,\;treated=1}\right)\\ & -\;\left({sentiment}_{time=1,\;treated=0}-{sentiment}_{time=0,\;treated=0}\right), \end{array}$$

where


$${sentiment}_{time=1,\;treated=1}=\beta_{0}+\beta_{1}+\beta_{2}+\beta_{3}$$

$${sentiment}_{time=1,\;treated=0}=\beta_{0}+\beta_{2}$$

$${sentiment}_{time=0,\;treated=1}=\beta_{0}+\beta_{1}$$

$${sentiment}_{time=0,\;treated=0}=\beta_{0}.$$

To highlight the risk of using arbitrary time windows with this type of data potentially showing substantially different effects, we use a variety of time windows around the start of the intervention: the full period of available data, 48 h around the intervention, 24, 12 and 1 h.

To assess the degree to which the findings from our DiD analyses may have been the result of random noise, *Bayesian causal impact analyses* were used as a convergent analytical method. This approach uses post-intervention control group data to estimate the counterfactual (i.e. that the intervention had no effect) based on the relationship between the treatment and control groups before the intervention. This allows for the calculation of pointive (the difference between observed treatment group values and the estimated counterfactual at a certain time point) and cumulative (the longitudinal sum of differences between observed treatment group values and the counterfactual) effects. Models were computed separately for original tweets and retweets for outcome variables of anger, fear and sadness (as the intervention was hypothesized to impact the sharing of content containing negative emotions; see above). Data were aggregated in 6 and 1 h increments, including the entire study length.

## 4. Results

### 4.1. Study 1

**Figure 2.**
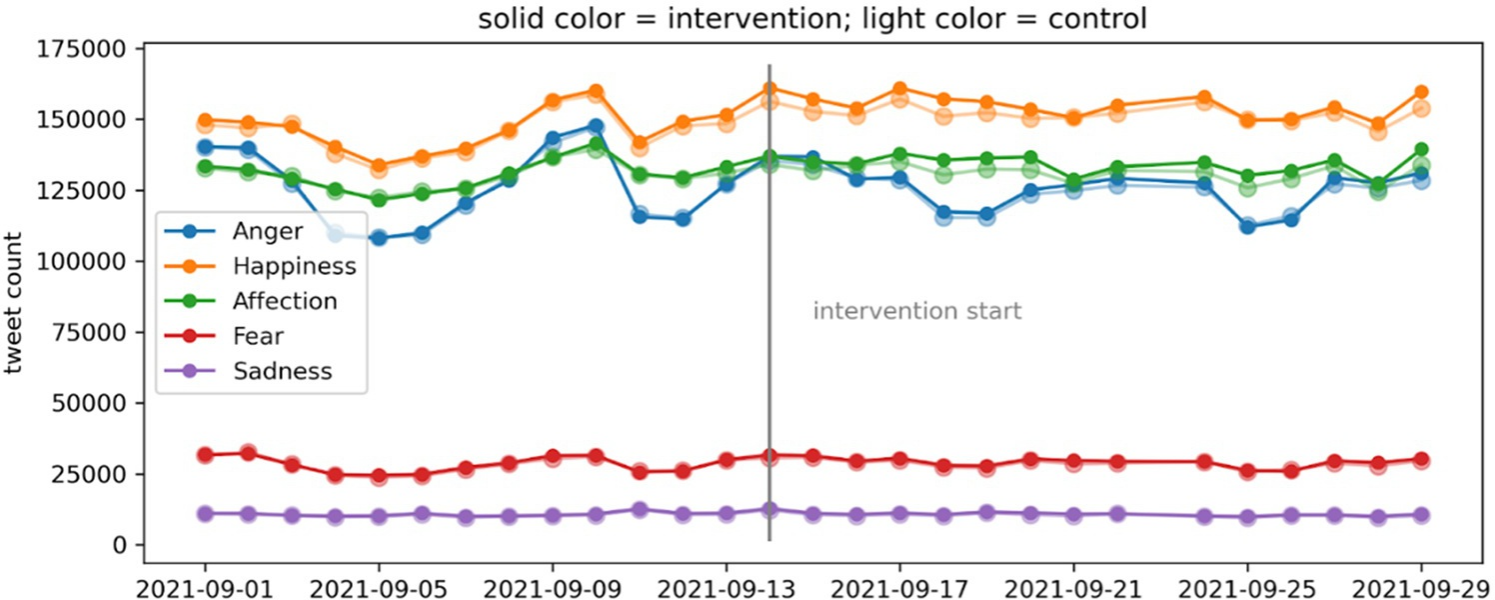
Tweet counts of tweets containing language related to anger, happiness, affection, fear and sadness, for the treatment and control groups.

Figure 2 shows the tweeting rates of language related to anger, happiness, affection, fear and sadness throughout the month of September 2021, for both the treatment and control groups. The figure shows no meaningful differences between both groups. The graph for retweets (rather than original tweets) shows very similar patterns (see electronic supplementary material, figure S1).

Next, we look at participants posting links to reliable and unreliable sources (as classified by a NewsGuard score of less than 60). Out of the 8 223 724 tweets with URLs posted by the treatment group, 39 055 tweets contained a link to a domain classified as ‘unreliable’ (± 0.47% of all tweets). Out of the 8 365 324 tweets with URLs posted by the control group, 39 361 tweets contained a link to a domain classified as ‘unreliable’ (also ±0.47%). In other words, we also do not find meaningful between-group differences with respect to how often Twitter/X users who were exposed to the inoculation video post misinformation (and/or other types of unreliable content).

Finally, we look at performance on the item-rating task in the follow-up survey. Contrary to our hypothesis and results reported by Roozenbeek *et al*. [20], we do not find that treatment group participants who filled in the follow-up survey are significantly better at discerning manipulative from neutral/non-manipulative social media content. We also do not find that they are more confident in identifying manipulation in social media content, nor do we observe an improvement in the quality of their self-reported sharing decisions (all *p*-values > 0.089, all BF₁₀ ~ 0; electronic supplementary material, table S1). However, this might be due to the fact that we only managed to collect a total of 68 valid responses, which is substantially lower than the approximately 1000 responses (500 participants per experimental condition) used by Roozenbeek *et al*. [20], as well as the ±120 participants per condition in Capewell *et al*. [30].

### 4.2. Study 2

**Figure 3.**
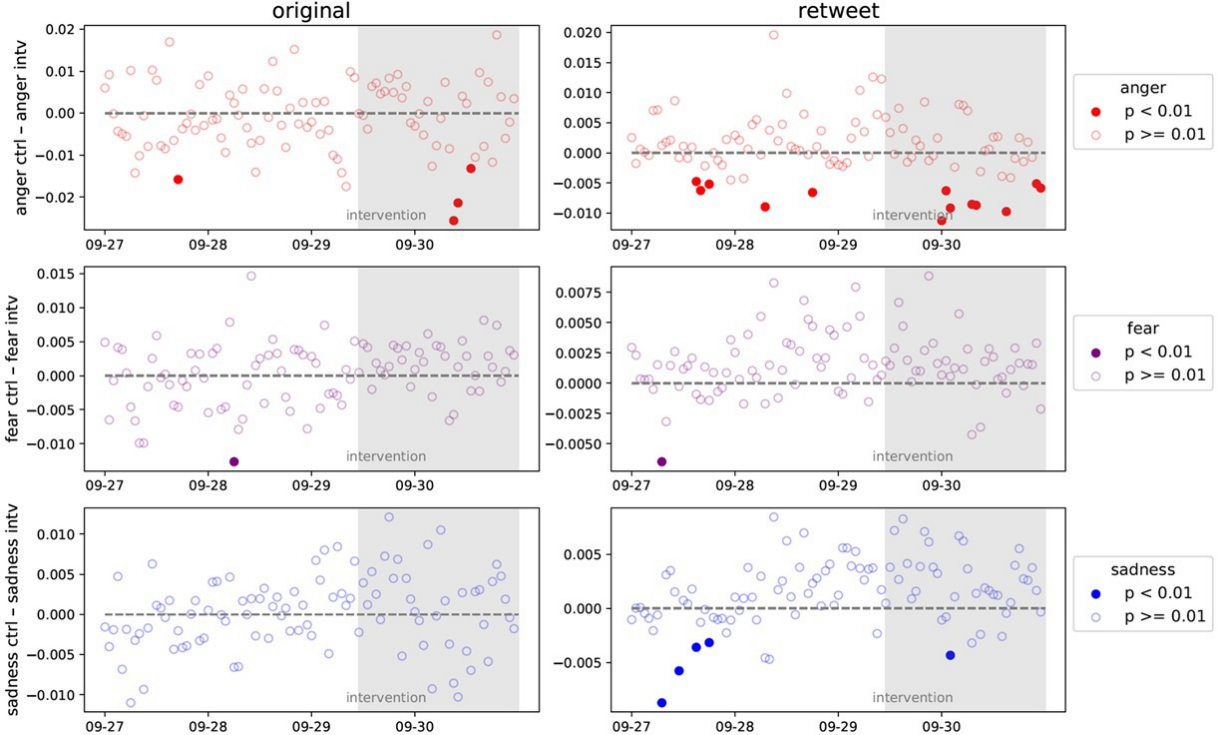
Mean differences per hour between the control and treatment groups for the use of anger, fear and sadness in original tweets and retweets, between 27 and 30 September 2022. Coloured dots indicate a significant between-group difference at the pre-registered alpha of 0.01. Grey zone indicates when the campaign is active. A negative difference indicates that the control group uses emotional language *less* than the intervention group, i.e. the results are *opposite* from the hypothesized direction. We also note that due to the large number of comparisons (4 days * 24 h per day * 3 distinct emotions * retweets/original tweets = 576), we would expect at least five comparisons to show a significant difference at *p* < 0.01 due to chance.

We plot the probability of the use of anger, fear and sadness (i.e. distinct emotions the sharing of which we predicted would be impacted by the intervention) in treatment and control group participants’ original tweets and retweets, between 27 September (2 days before the start of the ad campaign) and 30 September (2 days after the start; figure 3). Briefly put, we do not find evidence that watching an inoculation video leads to positive changes in people’s online behaviour. There also appears to be no significant changes in the content that people tweet themselves or what they retweet after the start of the campaign, and any effects that are significant are in the *opposite* direction to what we hypothesized (most likely due to sheer coincidence, as by random variation alone we would expect at least some effects to be significant at *p* < 0.01).

### 4.3. Supplementary analyses

**Figure 4.**
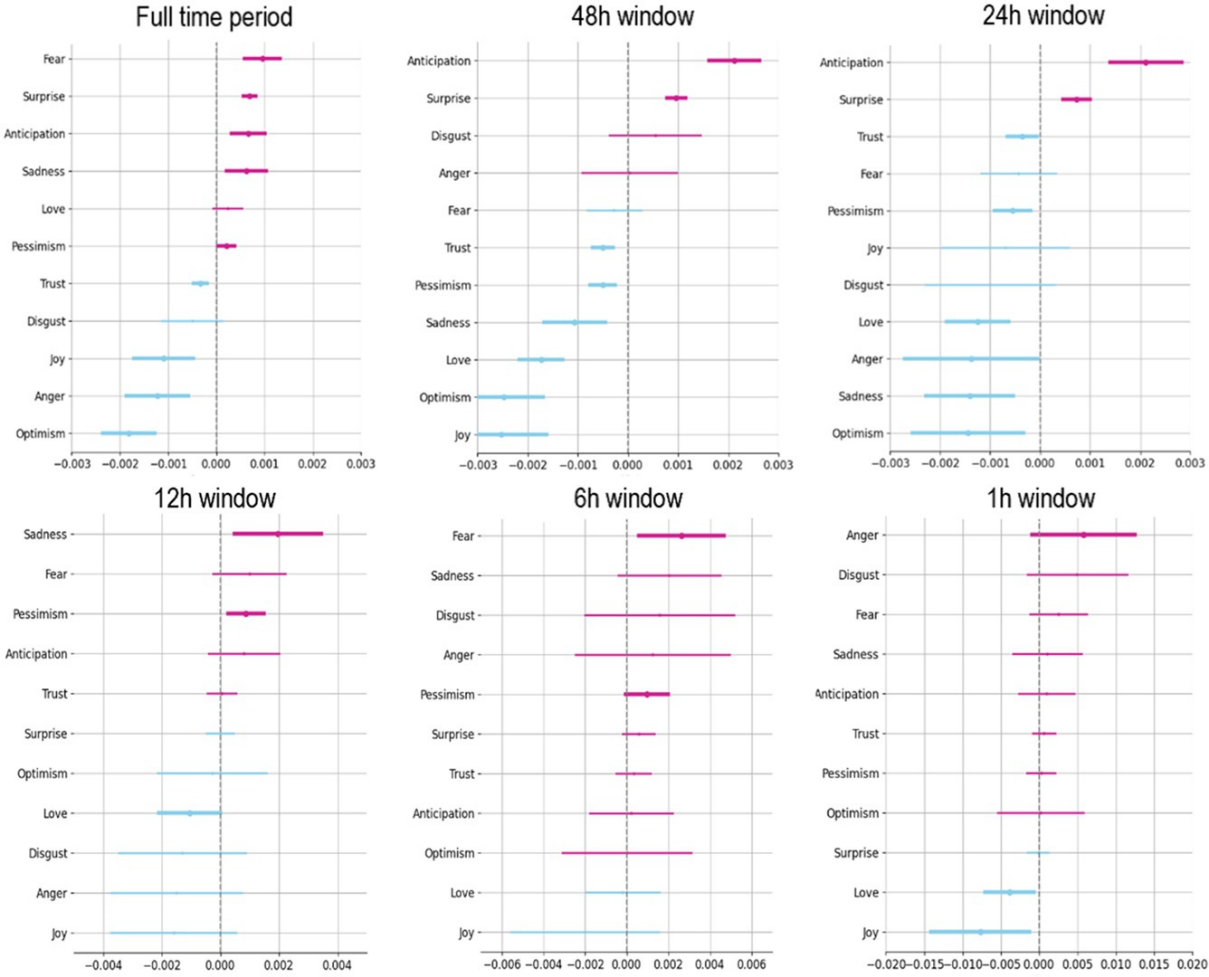
DiD analysis of retweets (treatment–control groups, before–after intervention), per sentiment and time window. A positive coefficient indicates a positive effect of the intervention on retweets of tweets containing that sentiment. Top left: whole time period of study. Top middle: 28 September 2022, 07.00 until 30 September 2022, 07.00 (24 h before–24 h after intervention start). Top right: 28 September 2022, 19.00 until 29 September 2022, 19.00 (12 h before–12 h after). Bottom left: 29 September 2022, 01.00 until 29 September 2022, 13.00 (6 h before–6 h after). Bottom middle: 29 September 2022, 04.00 until 29 September 2022, 10.00 (3 h before–3 h after). Bottom right: 29 September 2022, 06.00 until 29 September 2022, 08.00 (1 h before–1 h after). See electronic supplementary material, table S2, for coefficients and electronic supplementary material, figure S2 and table S3 for the results for a 30 min time window.

*DiD analysis*. Results are shown in figure 4 (see also electronic supplementary material, table S2).

Figure 4 shows significant between-group differences at *p* < 0.05 for the majority of sentiments included when looking at the entire time period our data includes (top left panel in figure 4); this is extremely unlikely to be the result of a true impact of the intervention, as prior research [30] has shown effect decay of this specific intervention within 48 h after being administered, even in terms of manipulation technique recognition (where the impact of the intervention is expected to be greater than for sharing behaviour). When zooming in on the 2-day period around the intervention (top middle and right panels), we again observe significant between-group differences in the retweeting of various kinds of sentiments, but these differences are not as hypothesized; the (negative) emotions specifically mentioned in the inoculation video (anger, fear, disgust) are not differentially retweeted between groups within this time period, and furthermore, both positive and negative sentiments are retweeted either more or less in an inconsistent manner (whereas we anticipated either a decrease in the retweeting of negative emotions, an increase in retweets of positive emotions, or both). When zooming in even further (to a 6, 3 and 1 h time window around the intervention start), we again see substantial changes both in the significance and the directionality of our effects (with effects disappearing almost entirely for the 1 hr period around the intervention). These further buttress our findings from study 1 and those shown in figure 3: based on our data, we cannot conclude that the interventions had a significant effect on social media sharing behaviour. Moreover, we urge caution with the use of arbitrary time windows when assessing intervention impact, which appears to be common in this field of research: depending on what time period is used as a cut-off, substantially different (significant) effects can be observed, *even in cases where the data is so noisy that a true effect is unlikely to be found*.

**Figure 5.**
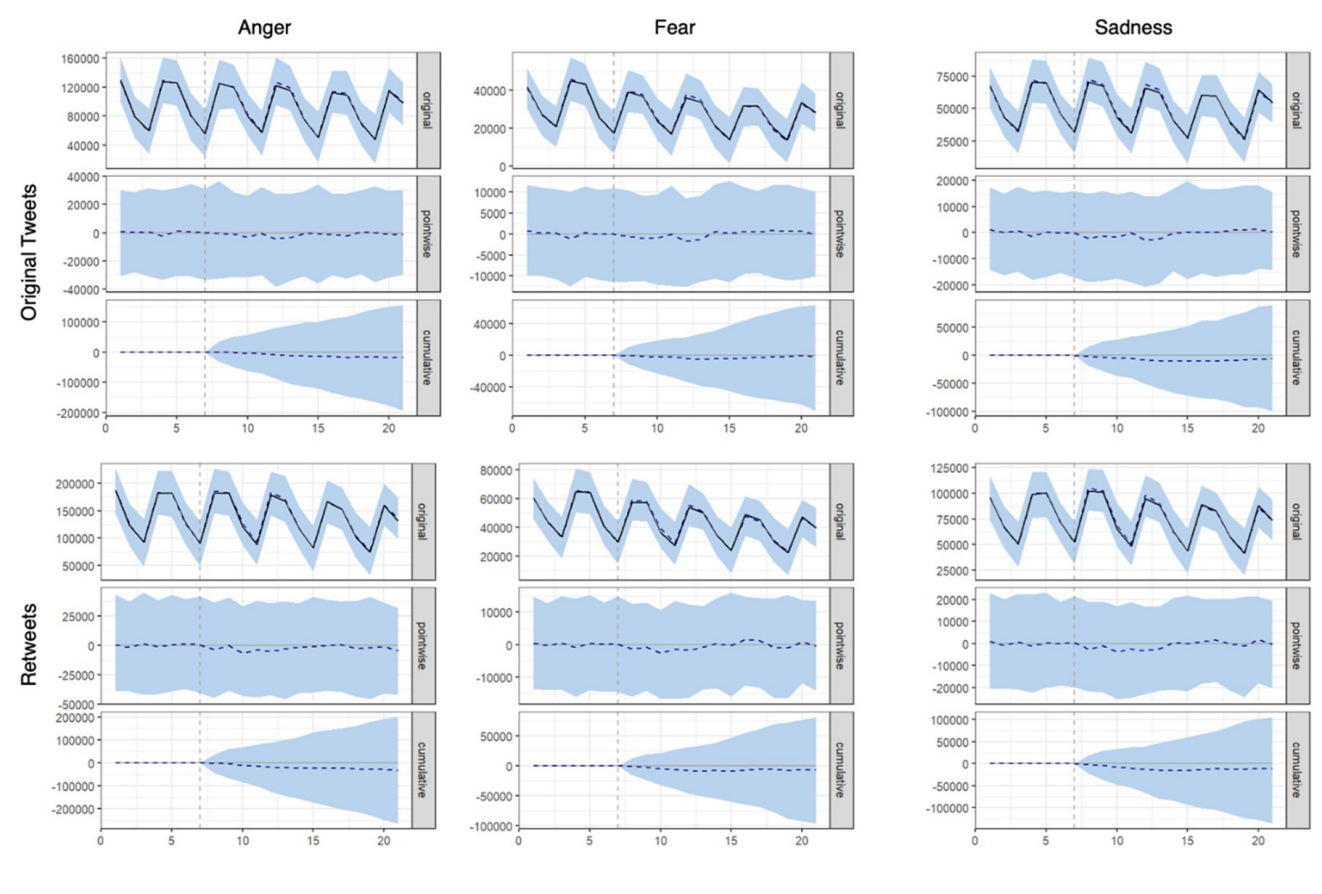
Bayesian causal impact analyses for tweets and retweets by emotions (anger, fear, sadness) throughout the study duration. Data aggregated at 6 h increments. Vertical grey line denotes intervention start time. A horizontal dotted line represents treatment group observed values. See electronic supplementary material, figure S3 for further details and electronic supplementary material, table S4 for the model output.

*Bayesian causal impact analysis*. Results from the Bayesian causal inference analysis are visualized in figure 5 (see also electronic supplementary material, table S4 for full model output). We found no support for a treatment effect on anger for original tweets (*B* = −0.011, s.d. = 0.071, 95% confidence interval (CI) = −0.14 to 0.14, *p* = 0.403) or retweets (*B* = −0.014, s.d. = 0.061, 95% CI = −0.13 to 0.12, *p* = 0.384), on fear for original tweets (*B* = 0.004, s.d. = 0.090, 95% CI = −0.14 to 0.20, *p* = 0.403) or retweets (*B* = −0.005, s.d. = 0.081, 95% CI = −0.14 to 0.18, *p* = 0.428), or sadness for original tweets (*B* = −0.004, s.d. = 0.066, 95% CI = −0.12 to 0.14, *p* = 0.446) or retweets (*B* = −0.009, s.d. = 0.055, 95% CI = −0.11 to 0.11, *p* = 0.405). This suggests that, indeed, the effects detected in the DiD analysis (figure 4) may have been false positives.

To investigate this possibility, additional causal impact models were computed with specifications more similar to the DiD analyses (specifically, that in frame C of figure 4) as possible. The average tweet emotion was computed in 1 h increments following Lin *et al*. [27], outcome variables as averages across total hourly posts and with the post-period truncated at 72 h post-intervention. Models were only computed for retweet data. These showed some significant effects, though these contradicted the DiD results from above. There was no treatment effect for fear (*B* = −0.005, s.d. = 0.009, 95% CI = −0.02 to 0.01, *p* = 0.281). However, there were small but significant *positive* effects of the treatment on retweets for both anger (*B* = 0.005, s.d. = 0.003, 95% CI = −0.02 to 0.01, *p* = 0.030) and sadness (*B* = 0.007, s.d. = 0.003, 95% CI = 0.00–0.01, *p* = 0.007). These results contradict our pre-registered hypotheses and the DiD analysis, which showed effects in the opposite direction. Taken together, these analyses highlight the risk of drawing false conclusions through significance testing, especially with noisy data and very small effect sizes. This further demonstrates the importance of convergent methods and analytical approaches: small differences in model specification—for instance, Bayesian versus frequentist estimations of the counterfactual, or different handling of missing values—can mean the difference between null and significant results, or even reverse the directionality of effects.

## 5. Discussion

This study is one of the first to explore how inoculation interventions aimed at countering misinformation can impact people’s behaviour on social media [31], focusing primarily on the overall quality of the content that people post and retweet on Twitter/X. Overall, we report null findings: we do not find evidence that the intervention (i.e. a 30 s video that ‘inoculates’ people against the use of emotionally manipulative language, validated and replicated in numerous previous studies) had a significant or substantial impact on people’s posting or retweeting behaviour. We offer several considerations.

First and foremost, our study suffered from a substantial methodological limitation, namely that Twitter/X uses (at least at the time our study was conducted) a so-called ‘fuzzy matching’ algorithm for targeted advertising. This means that of the lists of users we uploaded, only 30% were targeted with our ads, whereas the other 70% consisted of users that Twitter/X automatically matched to our user list. We therefore do not know which specific users were targeted with the ads to begin with. In other words, our study started out with 70% noise. Furthermore, not all users who *were* targeted actually watched the videos all the way through: as table 1 shows, approximately 25% of targeted users in the full study were actually counted as having seen the video. This means that a maximum of 7.5% of Twitter/X users we targeted (i.e. 25% of 30%) actually saw the video, but we do not know which 7.5%. These high levels of noise add substantial complications to our analyses and limit the strength of our conclusions. We were also unable to conduct a final follow-up study (which we had already pre-registered, see https://aspredicted.org/G6X_Y7P) due to an error on our Twitter/X Ads account. Specifically, the remaining funds available on our account disappeared overnight without warning from Twitter/X in February 2023. This meant that we were unable to explore whether our null findings may have been influenced by our Twitter/X ad settings (for instance, with respect to how often users are targeted with the video). We are therefore unable to claim a ‘true null’, that is, the true absence of an effect of inoculation interventions on social media behaviour. Instead, our findings are probably the result of treatment non-compliance (in the statistical sense, where condition assignment does not guarantee intervention receipt; see [34]). In other words, we cannot know how the intervention impacted participants’ sharing behaviour on Twitter/X, as we do not know which of our participants were treated with the intervention.

Our findings have several implications for how inoculation interventions, and behavioural interventions more broadly, are assessed in field studies. We chose the present intervention (an ‘inoculation’ video about emotional language) specifically because of its high degree of replicability: across several studies, the video was consistently shown to improve people’s accuracy in distinguishing emotionally manipulative from neutral social media content and improve the quality of their content sharing decisions, with a robust effect size and good longitudinal effects [16,20,30,31]. In addition, other video-based interventions, not based on inoculation theory, have shown null (or near-null) effects on behaviour in social media environments as well [25]. We therefore deem it unlikely that we obtained null results for our behavioural measures because the intervention was found wanting for efficacy in laboratory studies, or that inoculation theory is a poor framework for behavioural interventions compared with other frameworks (e.g. fact-checking videos).

Instead, we argue that difficulties in the implementation of our intervention, and the complications of doing so imposed by social media environments that are ill-designed for conducting field studies, are the primary explanation for our findings. Kane [35] lists seven alternative explanations (AEs) for null results in survey experiments. We discuss these explanations in the context of our findings in table 2. To summarize, it is likely that our findings are best explained by treatment non-compliance [34], rather than a substantive (‘true’) null.

**Table 2.** AEs for null findings in survey studies as discussed by Kane [35], and how they are relevant to the present study.

AEs	relevant?	explanation
1: respondents are inattentive to treatment	yes	approximately 25% of exposed respondents watched the video (table 1); this may not be enough to find an effect, as laboratory studies (e.g. [20]) ensured full treatment compliance prior to analysis.
2: failure to manipulate the independent variable of interest	no	the video has been extensively validated in previous studies and achieves its goal (boosting manipulation recognition + improving sharing intentions).
3: pre-treated respondents (respondents treated prior to study)	maybe	we used mutually exclusive lists of target Twitter/X users, but due to the ‘fuzzy matching’ policy, it is possible that control group users were targeted with the inoculation video and vice versa.
4: insufficient statistical power	maybe	sample sizes in this study were far larger than in laboratory studies (e.g. [20]); however, behavioural intervention effects are known to be small [25].
5: poor measurement of the dependent variable	yes	we used multiple indicators (retweets, original tweets, different emotions). However, we experienced substantial measurement error due to the Twitter/X ‘fuzzy matching’ policy (i.e. we do not know which users in our sample were targeted). Another possibility is that we used imperfect emotion/content classifiers, and higher-quality ones would have shown different effects.
6: ceiling or floor effects	maybe	most behaviours of interest are not rare in the sample (e.g. retweeting emotional content), but tweeting/retweeting of unreliable news is uncommon. Generally, sharing is a rare behaviour in online environments (relative to how much content users are exposed to).
7: countervailing/heterogeneous treatment effects	maybe	previous studies show little to no heterogeneity between groups (e.g. Liberals versus Conservatives [20]); however, other countervailing effects (e.g. algorithmic effects) cannot be fully ruled out.

Overall, we therefore argue that our findings speak more strongly to the limitations imposed by Twitter/X and more generally the complications of doing this type of research in real-world environments, rather than to the behavioural effects (or lack thereof) of inoculation interventions or informative videos. That said, there are several considerations around the behavioural effects (or lack thereof) of our and similar interventions.

How individual-level misinformation interventions affect people’s sharing behaviour relies on a series of assumptions that, to our knowledge, have only been discussed in one prior review paper [36]. These assumptions may help explain the overall small (and often non-existent) impacts of many different kinds of interventions aimed at fostering higher-quality sharing behaviour online. In order for any (individual-level) intervention, such as inoculation or nudges, to impact the sharing of unwanted content, it must first be the case that some social media users share unwanted content (such as misinformation) to begin with; this seems trivially true [37–39], although this group is known to be relatively small. Second, of this group of users who share unwanted content, some must do so out of ignorance or accidentally, without being highly motivated to do so (for example, for identity-defensive or political reasons). Recent research by Osmundsen *et al*. [40] indicates that although this group of users who share unwanted content out of ignorance or by accident exists, it is very small. Rather, most people appear to share misinformation out of partisan-political motivations. Another study by Ceylan *et al*. [41] found that misinformation sharing is to a large degree *habitual* as opposed to accidental, and this type of habitual sharing is less likely to be successfully reduced through the type of individual-level intervention used in the present study. Third, of the group of users that shares unwanted content unwittingly, a smaller group must be potentially amenable to any kind of intervention (i.e. they can be enticed to watch a video or play a game, be ‘nudgeable’, etc.; see [42]). Fourth, of this group, a yet smaller group must *actually* engage with the intervention as intended (e.g. they actually watch a video all the way through and learn the appropriate lessons from it, and so on [43]). Fifth, this group of users who are amenable to intervention must encounter unwanted content while the intervention is ‘active’, cognitively speaking; the effectiveness of all individual-level interventions decays over time, with time intervals ranging from potentially several seconds up to 1 h for implicit behavioural primes or one-off ‘accuracy prompts’ [27,44,45] to several days or weeks for more cognitively demanding interventions such as inoculation games and quizzes [16,26,30,46,47]. Sixth, of this group of amenable users who encounter unwanted content while the intervention is ‘active’, the intervention can only impact the content that these users *would have shared had it not been for the intervention* (in the sense that the intervention would obviously not impact unwanted content that these users would not have shared in the first place).

Therefore, regardless of whether individual-level interventions can impact content sharing in theory or in laboratory studies, it is reasonable to assume that the group of social media users who *actually* reduce their sharing of unwanted content as a direct result of the intervention is rather small [43]. This would explain why previous studies observed either no change or a very small change in sharing behaviour for a short duration [25,27–29]. One study that found a substantial behavioural impact of a single intervention for a good time duration (one week post-intervention) is the ‘spot the troll quiz’ study by Lees *et al*. [26], which also included an (exploratory) Twitter/X field study. However, this study reports a significant reduction in retweeting *overall*, in the sense that people who played their ‘spot the troll quiz’ retweeted less content *in total* during the first week after the intervention (compared with a control group which showed no such reduction). This finding may arise from an increased sense of uncertainty as to the reliability of online content, and thus increased carefulness in participants’ online behaviour. Whether this is a desirable outcome of the intervention is up for debate, but it is possible that it is too much to expect individual-level interventions to impact only the sharing of *unwanted* content (e.g. misinformation). System-level interventions (such as making changes to recommender algorithms; see [48]) must therefore also be considered, although these kinds of interventions potentially come with substantial downsides such as concerns about freedom of expression online [4,49]. Other research has called for looking at bottlenecks in the wider manipulation ecosystem, for example, by making manipulation (e.g. bot armies spreading misinformation) more expensive to create and maintain [50]. More research is needed to explore these questions in detail.

Relatedly, it is useful to discuss the potential benefits of individual-level misinformation interventions that seek to tackle the sharing of unwanted content versus those that seek to reduce the harmful effects of misinformation exposure (for example, by reducing belief in false claims, building resilience against manipulation, and so on). From our study, we can conclude that if an intervention has robust effects on the reduction of susceptibility to unwanted content as well as sharing *intentions* [20,30,31], this does not necessarily translate to robust and hypothesized changes in *actual* sharing behaviour. It is up for debate whether this is ‘good enough’ with respect to the effectiveness (real-world impact; as opposed to efficacy or successful results obtained in the laboratory; see [36]) of ‘boosting’ interventions which seek to foster a particular competence [51,52]. On the one hand, one could argue that successfully learning to identify false or misleading content should translate to predictable changes in behaviour (such as a reduction in the sharing of such content), and if this is not the case, then the intervention’s actual real-world impact could be seen as minimal. On the other hand, sharing any content at all on social media (including misinformation) is rare compared with how much content people see (i.e. people generally only share a small fraction of everything they see online), and so sharing-focused interventions can only ever seek to change these relatively rare behaviours. It is therefore possible that learning-based interventions such as the inoculation video we tested as part of this study have beneficial outcomes that cannot be measured as sharing behaviour, such as improved ‘critical ignoring’ [53]. This may be tested by looking at the impact of the intervention on outcomes such as video watch times (e.g. on YouTube).

Finally, using a multi-method robustness check procedure, we demonstrate the analytical complications of conducting ecologically valid intervention studies and highlight the very present risk of interpreting noise as signal. Because of Twitter/X’s ‘fuzzy matching’ policy and uncertainties as to whether participants who ‘viewed’ an ad (according to Twitter/X’s metrics) actually engaged with it as intended, our data contains a considerable amount of noise. Because we computed many analyses, some of our results were significant, but given the proportion of noise to signal, these results were probably the result of random chance. In cases like these, convergent methods of analysis (i.e. using multiple types of analyses to test the same question) can be useful to assess the robustness of results, but if (as was the case in our study) convergent methods show different effects, it can be highly risky to highlight the one method that shows the hypothesized effects. Moreover, our results emphasize how a study’s time frame can impact effects. As can be seen in figure 4, a small change in the time frame (narrowing the analysed window by a few hours) led to entirely different results. This can also be seen when comparing our results across analyses of the whole study period and those of a truncated period (figure 5 and electronic supplementary material, figure S3). For intervention studies to laud extremely short-term effects while trimming off later data that shows an effect decay or rebound effect is not, in our view, best practice. Rather, doing so creates a real risk of declaring success prematurely, which is made easier by the field’s current lack of rigour in theorizing intervention longevity prior to conducting field studies. At present, time windows in such behavioural studies are chosen arbitrarily. We therefore urge substantial caution in the interpretation of data collected from field studies, particularly when this data is noisy. We recommend a careful pre-emptive consideration of an appropriate analysis plan (including with respect to how quickly the intervention effect is expected to decay), pre-registering this plan and following it strictly after data collection.

## 6. Conclusion

This study has explored whether a previously validated ‘inoculation’ video can positively impact the quality of the content that people post and retweet on Twitter/X. After running two separate Twitter/X ad campaigns targeting a total of more than 900 000 users who had previously shared misinformation, we do not find evidence for any substantial behavioural change in terms of what people share and retweet. This is in large part due to Twitter/X’s ‘fuzzy matching’ policy, which introduced substantial noise in our datasets. We therefore argue that our findings primarily represent a treatment non-compliance null, rather than a ‘true’ null effect of inoculation interventions on sharing behaviour. A compounding problem was that we were unable to conduct our final follow-up study due to our funds disappearing from our Twitter/X Ads account. We further demonstrate that convergent analyses and arbitrary time frames (e.g. looking at intervention effects over 1 h versus 6 or 24 h) can yield contradictory (yet still significant) effects, and note a substantial risk of interpreting noise as signal, and call for the rigorous, theory-based pre-registration of the longitudinal testing of interventions in field study designs.

Finally, we argue that large-scale behavioural change is possibly too much to expect from relatively light-touch individual-level interventions such as ‘inoculation’ videos, as the target group that would potentially benefit from such an intervention in terms of improving the quality of the content they share may simply be too small to yield a detectable effect using our currently available methods. Because only a small subset of people who are exposed to misinformation (and may believe it or be otherwise influenced by it) share it in any kind of measurable way, sharing-focused interventions may be overall less impactful than interventions that reduce misinformation susceptibility (i.e. interventions that mitigate the potentially harmful effects of exposure to misinformation).

## Data Availability

Both studies were pre-registered (study 1: https://aspredicted.org/9NX_CYQ, study 2: https://aspredicted.org/YXR_FBN). All data, item sets and supplementary analyses necessary to replicate our findings can be found on our OSF page: https://osf.io/3whuy/. We report how we determined our sample size, all data exclusions, all manipulations and all measures in the study. Supplementary material is available online [54].

## Footnotes

^1^
We had also planned to conduct follow-up studies (e.g. see https://aspredicted.org/G6X_Y7P), but these were compromised by Elon Musk’s takeover of Twitter/X in October of 2022; for instance, due to substantial changes in staff, including in the advertising department, we could not get a hold of our contacts for several months.

## Authors' Notes

Informed consent was obtained from survey study participants (Study 1). No informed consent could be obtained from Twitter/X users exposed to the intervention(s), but we only used publicly available Twitter data available through the company’s API, in line with common practices in social media research.
